# S02-2 Socioeconomic determinants of physical activity, sleep and screen time among children aged 6-9 years of age in Europe

**DOI:** 10.1093/eurpub/ckac093.007

**Published:** 2022-08-29

**Authors:** Sanja Music Milanovic, Marta Buoncristiano, Helena Križan, Giulia Rathmes, Julianne Williams, Jolanda Hyska, Vesselka Duleva, Hana Zamrazilová, Tatjana Hejgaard, Maja Bæksgaard Jørgensen, Benoît Salanave, Lela Shengelia, Cecily C Kelleher, Angela Spinelli, Paola Nardone, Shynar Abdrakhmanova, Zhamilya Usupova, Iveta Pudule, Ausra Petrauskiene, Victoria Farrugia Sant'Angelo, Enisa Kujundžic, Anna Fijałkowska, Ana Isabel Rito, Alexandra Cucu, Lacramioara Aurelia Brinduse, Valentina Peterkova, Andrea Gualtieri, Marta García-Solano, Enrique Gutiérrez-González, Khadichamo Boymatova, Mahmut S Yardim, Maya Tanrygulyyeva, Marina Melkumova, Daniel Weghuber, Eha Nurk, Päivi Mäki, Ingunn Holden Bergh, Sergej M Ostojic, Kenisha Russell Jonsson, Igor Spiroski, Harry Rutter, Wolfgang Ahrens, Ivo Rakovac, Stephen Whiting, João Breda

**Affiliations:** 1 Croatian Institute of Public Health, Zagreb, Croatia; 2 School of Medicine, University of Zagreb, Zagreb, Croatia; 3 Division of Country Health Programmes, WHO Regional Office for Europe, Copenhagen, Denmark; 4 Nutrition and Food Safety Sector, Institute of Public Health, Tirana, Albania; 5 Department Food and Nutrition, National Centre of Public Health and Analyses, Sofia, Bulgaria; 6 Obesity Management Centre, Institute of Endocrinology, Prague, Czech Republic; 7 Health Promotion and Inequality, Danish Health Authority, Copenhagen, Denmark; 8 National Institute of Public Health, University of Southern Denmark, Odense, Denmark; 9 Department of Non-Communicable Diseases and Traumatisms, Santé Publique France, the French Public Health Agency, Saint-Maurice, France; 10 Nutritional Surveillance and Epidemiology Team (ESEN), University Sorbonne Paris Nord, Bobigny, France; 11 Maternal, Child and Reproductive Health, National Center for Disease Control and Public Health, Tbilisi, Georgia; 12 College of Health and Agricultural Sciences, University College Dublin, Dublin, Ireland; 13 National Centre for Disease Prevention and Health Promotion, Italian National Institute of Health (Istituto Superiore di Sanità), Rome, Italy; 14 Department of Science and Professional Development, National Center of Public Health of the Ministry of Health of the Republic of Kazakhstan, Almaty,Kazakhstan; 15 Kazakhstan School of Public Health, Kazakhstan's Medical University, Almaty, Kazakhstan; 16 Republican Center for Health Promotion and Mass Communication, Ministry of Health of the Kyrgyz Republic, Bishkek, Kyrgyzstan; 17 Department of Research and Health Statistics, Centre for Disease and Prevention Control, Riga, Latvia; 18 Department of Preventive Medicine, Lithuanian University of Health Sciences, Kaunas, Lithuania

**Keywords:** Screening time, screen-based activities, sleeping behaviour, family record form, parental questionnaire

## Abstract

**Background:**

Physical activity is key for preventing obesity and development of noncommunicable diseases later in life. Previous research suggests that socioeconomic factors, such as parental education or income, may influence a child’s risk of obesity. However, previous research on this has provided heterogeneity in results. Our aim was to investigate the socioeconomic disparities between physical activity, sedentary behaviour and sleep patterns in school-aged children aged 6 to 9 years in 24 European countries, using a large nationally-representative sample of children from 24 countries (Albania, Bulgaria, Croatia, Czechia, Denmark, France, Georgia, Ireland, Italy, Kazakhstan, Kyrgyzstan, Lithuania, Latvia, Malta, Montenegro, Poland, Portugal, Romania, Russian Federation – only Moscow, San Marino Republic, Spain, Tajikistan, Türkiye and Turkmenistan).

**Methods:**

COSI collected information on physical activity patterns of children, sedentary behaviour and sleep duration through a questionnaire filled by parents. Among these, the paper focused on the following behaviours: Transportation to and from schools, Time spent on practising sports, Time spent on actively/vigorously playing, Time spent watching TV or using electronic devices and Hours of sleep per night. For the paper purpose, countries were grouped in 4 macro-regions according to United Nations “Standard Country or Area Codes for Statistical Use”.

**Results:**

Findings indicated that a high prevalence of motorized school transport among children of employed parents in Southern Europe. The highest prevalence of insufficient sports and active play was among families from West-Central Asia who meet the end of the month with troubles, the highest prevalence of excessive screen time is among families from Eastern Europe, where both parents have a low level of education and the highest prevalence of insufficient sleep is among families from West-Central Asia where both parents have a high level of education.

**Conclusions:**

There are important differences in the socioeconomic determinants of PA, sleep and screen related behaviours both between countries and sub-regions across the WHO European Region. This analysis of results from the COSI survey provides important insights that can help guide policy makers to take action to address the childhood obesity epidemic.

